# Dietary Probiotic Supplementation Suppresses Subclinical Necrotic Enteritis in Broiler Chickens in a Microbiota-Dependent Manner

**DOI:** 10.3389/fimmu.2022.855426

**Published:** 2022-03-18

**Authors:** Ying Zhao, Yan Zeng, Dong Zeng, Hesong Wang, Ning Sun, Jinge Xin, Mengjia Zhou, Hanbo Yang, Lei Lei, Hongli Ling, Abdul Khalique, Danish Sharafat Rajput, Baoxing Gan, Zhiqiang Wan, Zhipeng Yao, Jing Fang, Kangcheng Pan, Gang Shu, Bo Jing, Dongmei Zhang, Xueqin Ni

**Affiliations:** ^1^ Animal Microecology Institute, College of Veterinary, Sichuan Agricultural University, Chengdu, China; ^2^ Guangdong Provincial Key Laboratory of Gastroenterology, Department of Gastroenterology, Institute of Gastroenterology of Guangdong Province, Nanfang Hospital, Southern Medical University, Guangzhou, China; ^3^ Sichuan Academy of Animal Sciences, Animal Breeding and Genetics Key Laboratory of Sichuan Province, Chengdu, China; ^4^ Chengdu Slan Biotechnology Co., Ltd, Chengdu, China; ^5^ Qingdao Vland Biotech Inc, Qingdao, China

**Keywords:** probiotics, subclinical necrotic enteritis, broiler chickens, gut microbiota, gut–liver axis, poultry production

## Abstract

**Background:**

Chicken meat is one of the most consumed meats worldwide and poultry production is increasing at an exponential rate. Reducing antibiotic usage has resulted in the recurrence of subclinical necrotic enteritis again and influenced global poultry production. Probiotics are potential antibiotic substitutes that can be used to prevent subclinical necrotic enteriti. However, the precise mechanism of action of probiotics and information on which gut microbes confer this efficacy remain elusive.

**Methods and results:**

The subclinical necrotic enteritis animal model was used to reveal the mechanism underlying the effect of probiotics on intestinal health through RNA sequencing and 16S rDNA amplicon sequencing. *Bacillus licheniformis* H2 feeding significantly reduced the relative abundance of *Clostridium perfringens* in the ileum and markedly ameliorated the pathological damage in the ileum and liver. In addition, oral administration of *B. licheniformis* H2 contributed to the enhancement of the intestinal barrier function and epithelial renewal, reducing energy consumption, and improving enteral nutrition absorption. Probiotic *B. licheniformis* H2 also ameliorated the inflammatory response and increased the immunity of subclinical necrotic enteritis infected broilers. Finally, *B. licheniformis* H2 feeding regulated liver gene expression to suppress immune response and promoted growth and metabolism depending on the gut microbiota.

**Conclusions:**

These results indicated the mechanism of probiotic action of *B. licheniformis* H2 in maintaining intestinal health and thus promoting growth and *B. licheniformis* H2 may serve as an antibiotic substitute to prevent subclinical necrotic enteritis in poultry farming.

## Introduction

Chicken is a major source of animal protein for humans (1). However, necrotic enteritis (NE), an enteric bacterial disease in poultry industry, significantly impacts the attempts to increase global poultry production. The annual loss in the global economy due to NE is estimated to exceed $6 billion (2). Acute or clinical NE is characterized by sudden death of up to 50% mortality, whereas the more prevalent subclinical form of NE (SNE) showed neither peak mortality nor overt clinical symptoms (3–5). Therefore, SNE-infected individuals are usually difficult to detect, thereby causing more considerable economic and profitability losses (5, 6). *Clostridium perfringens* (CP) is the main causative pathogen of SNE. Its infection in chickens could trigger food-borne diseases in humans (7). The occurrence and development of SNE depends on predisposing factors and the presence of pathogenic *C. perfringens* strains (8, 9). *Eimeria* infection, a major predisposing factor for SNE, provides a rich nutrient source for supporting CP proliferation by impairing the intestinal barrier (8, 9). Other predisposing factors (e.g., diet and stress) can also change the intestinal environment to provide a more favorable growth advantage to *C. perfringens* (9–11). Virulence factors (e.g., alpha, beta, and NetB toxins) are produced by pathogenic *C. perfringens* strains; these factors, lead to gross damage of the epithelia (10–12).

Poultry production and nutrition are interlinked with intestinal health. Crucial functions of the intestinal barrier include maintaining intestinal health, allowing the absorption of nutrients, and limiting the entry of potential pathogens, thereby resulting in the optimal performance and health of broiler chickens (13, 14). The disruption of the intestinal barrier can lead to “leaky gut” and nutrient malabsorption (14, 15). This intestinal barrier dysfunction exposes the liver to bacterial components and metabolites and gut bacteria, thereby leading to liver inflammation (14–16). *C. perfringens* colonizes the liver through the portal vein during SNE infection (12, 17, 18). Cholangiohepatitis is induced by a large number of *C. perfringens* in the liver (12, 17, 18), which indicates that the gut–liver axis, which is the close functional and vascular association between gut and liver, is closely related to SNE pathogenesis.

Antibiotics have been widely used to control SNE to increase poultry production. However, the mounting concerns on the development of resistance in bacteria prompted the ban or restriction of antibiotic usage in many regions, leading to the outbreak of SNE again (19, 20). In recent years, the interest in the use of alternatives to antibiotics has surged. Probiotics reportedly improve nutrient digestibility and the overall health of the host, which means they are ideal substitutes for antibiotics (21). The beneficial mechanism of probiotics is the modification of the composition of intestinal microflora to restore and maintain intestinal homeostasis (22–24). Many studies have shown that healthy intestinal microbiota is important. It has remarkable implications for immunity, inflammation, energy metabolism, nutrient availability and absorption rate, and productivity in broiler chickens (25, 26). Birds infected with SNE have an altered gut microbiota profile, such as reduced abundance of *Firmicutes*, *Lactobacillus*, and *Bacteroides*, which were associated with healthy status and high productivity in chickens (6). Manipulating gut microbiota *via* the use of probiotics is a promising strategy for controlling SNE. However, the precise mechanism of probiotics and which gut microbes confer this efficacy remain largely unclear.

Therefore, this study was conducted to investigate the underlying mechanisms of probiotics involved in shaping the gut microbiota and in reducing the negative influence of SNE on gut health through the gut–liver axis. Probiotics isolated from the intestine of the host itself are more effective compared with those from other sources, because local microorganisms colonize and stabilize the gut more easily (27, 28). The natural probiotic *Bacillus licheniformis* H2 (H2) originally isolated from the ileum of healthy chickens was utilized in this study. H2 can exert a positive effect on the growth performance of broiler chickens damaged by SNE, of which the mechanism may be related to intestinal development, lipid anabolism, antioxidant capacity, and apoptosis which were improved by H2 (29, 30).

## Methods

### Animal Ethics Statement

All animal experiment procedures in this study were conducted in accordance with the guidelines of the Animal Welfare Act and all procedures and protocols were approved by the Institutional Animal Care and Use Committee of the Sichuan Agricultural University (approval number: SYXKchuan2019-187). All efforts were made to minimize the suffering of the animals.

### Animals and Diets

One-day-old male broiler chicks (n = 180) were purchased from a commercial hatchery (a local broiler hatchery in Chengdu, China). The animals had the same genetic background (Ross 308 strain) and similar birth weights. Chicks were reared in a controlled room (relative humidity, 60–70%; room temperature, maintained at 33°C for the first 3 days and subsequently decreased by 3°C every week up to a final temperature of 24°C; lighting, 24 h daily). Birds had free access to water and food. An antibiotic-free and standard broiler diet mostly consisted of corn and soybean meal; it was formulated according to the NRC. The detailed composition of the basal feed and nutrient levels are shown in **Appendix Table S1**.

### Experimental Design and Sample Collection

**Figure 1:**
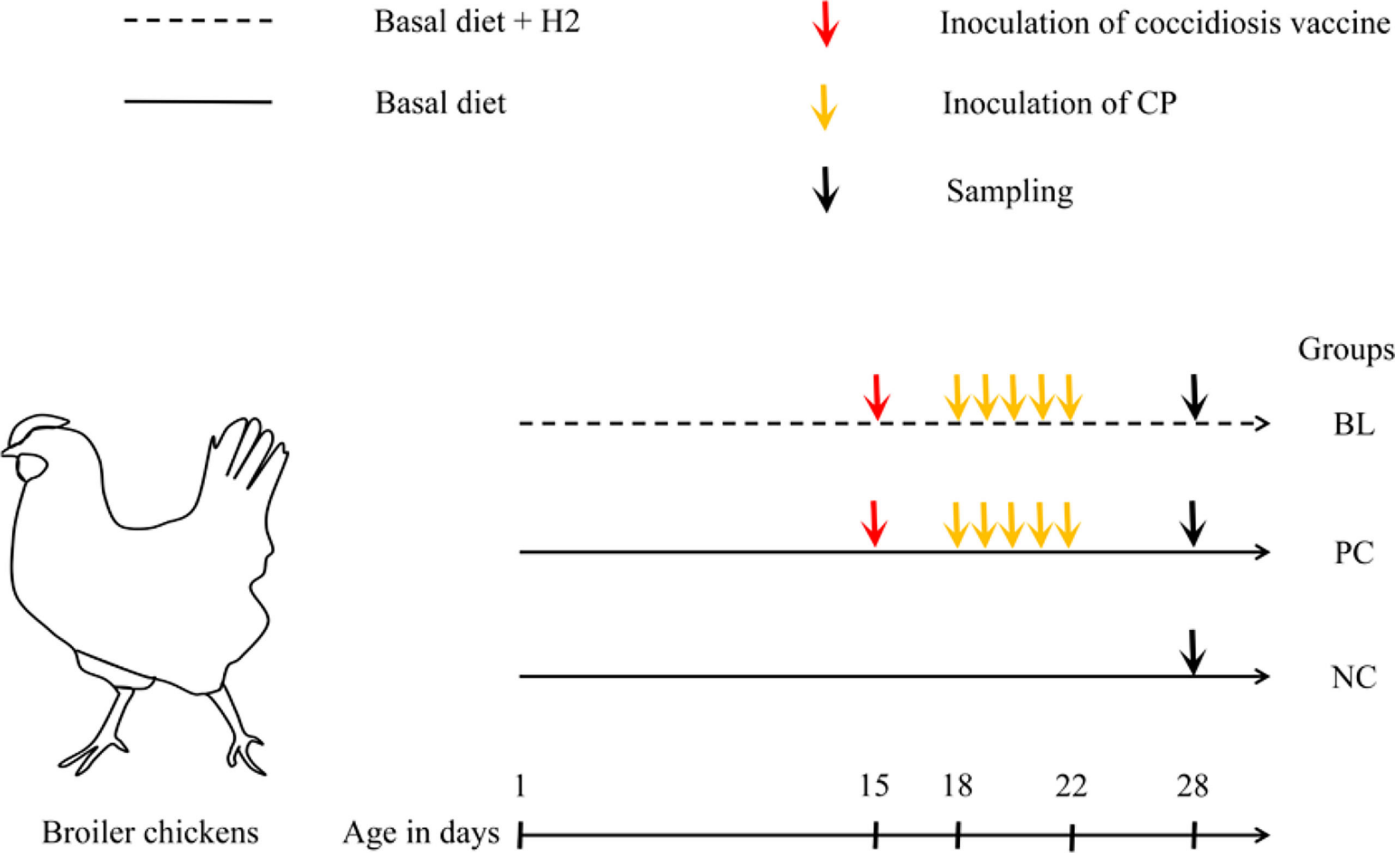
Sketch of animal experiment design. Newly hatched chicks with similar birth weights were randomly allocated to three groups with different treatments. NC, birds received basal diet supplemented with LB liquid medium; PC, birds received basal diet and induction of SNE; BL, birds received induction of SNE and basal diet supplemented with 1.0 × 10^6^ cfu *B licheniformis* H2/g diet.

One-day-old male chicks were weighed and randomly divided into three groups. Each group had six replicates with 10 birds per replicate. Each replicate chick was reared in the same cage. The experimental groups were as follows (**Figure 1**): (i) a negative control group (NC); (ii) a positive control group infected with SNE (PC); and (iii) a preventing group infected with SNE and supplemented with *B. licheniformis* H2 (BL). Birds in NC and PC groups were fed basal diets plus LB liquid medium. Birds in BL group were fed basal diets plus probiotic *B. licheniformis* H2 at 1.0 × 10^6^ CFU/g feed. On day 15, birds in the PC and the BL groups were orally inoculated with a 10-fold dose of live coccidiosis vaccine. Subsequently, the birds were orally challenged with CP (1 ml, 2.2 × 10^8^ CFU/ml) daily from 18 to 22 days of age.

Before the end of the experiment, one bird from each replicate was randomly selected, and weighed after fasting for 12 h, and executed by jugular vein bloodletting on the 28th day. Blood samples were collected from the jugular vein, incubated at 37°C for 2 h. Serum samples were separated by centrifuging the blood at 2,000×*g* for 15 min and storing at −30°C. Thymus gland (on the right side), bursal, spleen, and liver were removed simultaneously from each chicken and weighed. Two liver samples were collected. One was washed with ice-cold phosphate buffered saline (PBS) and then fixed with fresh 4% paraformaldehyde for pathological detection. The other one was rapidly frozen in liquid nitrogen and stored at −80°C for transcriptome detection. The ileum tissue (about 1–2 cm) was removed, gently washed with cold PBS, and then fixed for paraffin embedding. Additionally, ileum and cecum contents were collected and frozen at −80°C for microbial flora detection.

### Bacterial Strains and Coccidiosis Vaccine

The *B. licheniformis* H2 strain (CCTCC NO: M2011133) was originally isolated from the ileum of healthy chickens by the animal microecology research center of the Sichuan Agricultural University. The *C. perfringens* type-A strain (CVCC2030) was purchased from the China Veterinary Culture Collection Center, isolated from the intestines of broiler chickens with necrotic enteritis. The culture and use of H2 and CP were based on our previous study (29). The DLV coccidium vaccine was provided by the Shanghai Veterinary Research Institute, Chinese Academy of Agricultural Sciences.

### Histological Examination of the Ileum and Liver Tissues

The ileum and liver tissues of each chicken were fixed in 4% paraformaldehyde solution for 1 week, processed, trimmed, and embedded in paraffin. Tissue sections (5 μm in thickness) were prepared from tissue blocks with a microtome and stained with hematoxylin and eosin. All the prepared tissue slides were histologically examined using a light microscope (Olympus, Japan).

### Measurement of Organ Index

One broiler randomly selected from each replicate in each group was weighed and sacrificed for the measurement of organ index. Thymus gland (on the right side), bursal, spleen, and liver tissues were removed. Organ index was calculated by using the following equation:

Organ index = organ weight (mg)/live body weight (g) × 100%.

### Detection of Immunoglobulins and Cytokines of the Ileum, Liver, and Serum

The concentrations of immunoglobulins (IgA, IgG, and IgM) in the liver, serum, and ileum were measured with a sandwich ELISA using microtiter plates and chicken-specific IgA, IgM, and IgG ELISA quantitation kits (Shanghai Enzyme-linked Biotechnology Co., Ltd., Shanghai, China). The ELISA procedure was executed strictly in accordance with the instructions of the manufacturer. Absorbance was measured at 450 nm using a microplate autoreader (Thermo Lab System, Helsinki, Finland). The levels of cytokines, namely, interleukin (IL)-2, IL-4, IL-6, IL-8, IL-10, IL-17, interferon-gamma (IFN-γ), and tumor necrosis factor-alpha (TNF-α), in the liver, serum, and ileum were determined by chicken-specific ELISA kits (Shanghai Enzyme-linked Biotechnology Co., Ltd., Shanghai, China).

### Microbial Genomic DNA Extraction

Total DNA extraction from ileum and cecum contents was performed in accordance with the procedure of the Omega E.Z.N.A.TM Stool DNA Isolation kit (Omega Bio-tek, GA, USA). DNA purity and integrity were determined by agarose gel electrophoresis (AGE). DNA concentration was precisely quantified using an Invitrogen Qubit 4 Fluorometer (Thermo Fisher Scientific Inc., Waltham, MA, USA). DNA was stored at −20°C until further processing.

### Bacterial 16S rDNA Gene Sequencing

The bacterial V4 hypervariable region within the 16S rRNA gene was amplified by using broadly conserved primer pairs 515F (5′-GTGCCAGCMGCCGCGGTAA-3′) and 806R (5′-GACTACHVGGGTWTCTAAT-3′). PCR reactions (30 µl) consisting of 15 µl of Phusion^®^ High-Fidelity PCR Master Mix (New England Biolabs), 0.2 µM of forward and reverse primers, and about 10 ng template DNA were conducted. The PCR amplification conditions were as follows: initial denaturation at 98°C for 1 min, 30 cycles of denaturation at 98°C for 10 s, annealing at 50°C for 30 s, elongation at 72°C for 30 s, and a final extension step at 72°C for 5 min. The amplified products were detected using 2% agarose gel, and further purified with GeneJET™ Gel Extraction Kit (Thermo Fisher Scientific Inc., Waltham, MA, USA) in accordance with the recommendations of the manufacturer, and quantified by using a Qubit 2.0 fluorometer (Thermo Fisher Scientific Inc., Waltham, MA, USA). At last, the purified amplicons were sequenced on the Illumina MiSeq 2 × 250 platform conducted by the Novogene Company (Beijing, China).

### Sequence Processing and Bioinformatics Analysis

Quality filtering was performed by using Cutadapt software (V1.9.1, http://cutadapt.readthedocs.io/en/stable/) according to the method introduced by Martin. Then, the filtered sequences were aligned with the reference database (Silva database, https://www.arb-silva.de/) using the UCHIME algorithm (UCHIME Algorithm,

http://www.drive5.com/usearch/manual/uchime_algo.html) to remove chimera sequences. The high-quality sequences were analyzed by Uparse software (Uparse v7.0.1001, http://drive5.com/uparse/). Sequences were assigned to the same operational taxonomic units (OTUs) based on 97% sequence similarity. A representative sequence for each OTU was screened for further annotation based on the Silva Database (https://www.arb-silva.de/). Alpha diversity indices namely, Observed-species, Chao1, Shannon, Simpson, ACE, and Good-coverage, were calculated by QIIME (Version1.7.0) and drawn using R software (Version 2.15.3). Beta diversity analysis based on weighted unifrac were calculated with QIIME software (Version 1.7.0) and displayed by principal coordinate analysis (PCoA). Relative abundances of phyla, classes, orders, families, genera, and species were statistically compared among the different groups. Linear Discriminant Analysis Effect Size (LEfSe) analysis was conducted using LEfSe software.

### Transcriptome Analysis of Liver Tissue

The total RNA from liver samples was extracted using an RNAiso Plus reagent (TaKaRa, Dalian, China) in accordance with the protocol of the manufacturer. RNA samples with RNA integrity number (RIN) value ≥7 were used for library construction (Agilent, CA, USA). RNA-seq libraries were prepared using the TruSeq Stranded mRNA LT Sample Prep Kit according to the specifications (Illumina, San Diego, USA) and then sequenced using Illumina HiSeq™ 2500 by the Genedenovo Biotechnology Co., Ltd. (Guangzhou, China).

### The Gene Expression and Abundance of *C. perfringens*

The total RNAs of liver and intestinal samples (namely, jejunum and ileum) were extracted separately using the EZNA^®^ Total RNA Isolation Kit (Omega Bio-tek, GA, USA) in accordance with the instructions of the manufacturer. The synthesis of the first strand (cDNA) was performed using the RevertAid First Strand cDNA Synthesis Kit (Thermo Fisher Scientific Inc., Waltham, MA, USA) in accordance with the instructions of the manufacturer. RT-qPCR reactions were conducted using prepared cDNA products of liver and intestinal samples. The reactions were conducted in a CFX96 Real-Time PCR Detection System (Bio-Rad, Hercules, CA, USA). The relative expression of genes was normalized to GAPDH and calculated using the 2^−ΔΔCt^ method (31). The gene expression of the *C. perfringens* was normalized using the 16S rRNA gene and calculated using the 2^−ΔCt^ method in accordance with Huang et al. (32). The primer sequences used for the gene expression analysis and bacterial abundance analysis are shown in **Appendix Table S2**. The cycling conditions were as follows: 95°C for 10 min, followed by 40 cycles of 95°C for 30 s, 52°C for 30 s, and 72°C for 1 min (32).

### Host Transcriptome–Microbiome Correlation Analysis

The Pearson’s correlation between liver transcriptome and gut microbiome was calculated using R software (Version 2.15.3).

### Statistical Analysis

Statistical analysis of immune organ index, immunoglobulins, cytokines, and relative expression of genes were performed by one-way ANOVA followed by Duncan’s multiple comparison tests. Results were statistically significant if *P*-values were <0.05.

## Results

### Probiotic H2 Suppresses Proliferation of CP and SNE-Induced Inflammation in the Broiler Chickens

**Figure 2:**
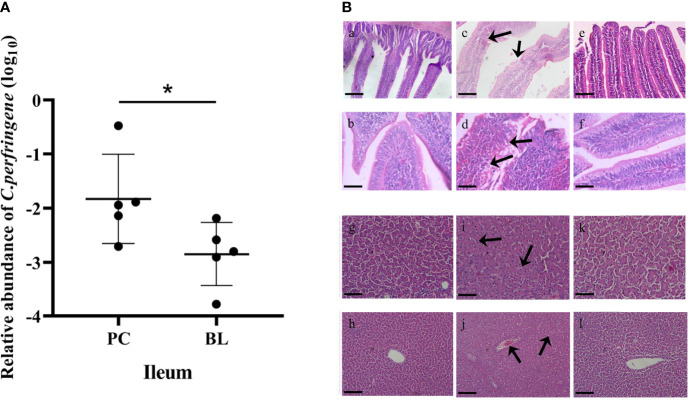
Probiotic H2 suppresses proliferation of CP and SNE-induced inflammation in broiler chickens. **(A)** Relative abundance of *C. perfringens* in the ileum. *P <0.05, n = 6, measured using ANOVA. **(B)** Hematoxylin–eosin (H&E) staining in the ileum and liver. Scale bar: 2 mm. **(A–F)** Images of ileal sections. **(A)** NC group (magnification × 200); (**B**) NC group (magnification × 400); **(C)** PC group (magnification × 200); **(D)** PC group (magnification × 400); **(E)** BL group (magnification × 200); **(F)** BL group (magnification × 400). **(G–L)** Images of liver sections. **(G)** NC group (magnification × 200); **(H)** NC group (magnification × 400); **(I)** PC group (magnification × 200); **(J)** PC group (magnification × 400); **(K)** BL group (magnification × 200); **(L)** BL group (magnification × 400).

The inhibitory effect of H2 on *C. perfringens* was evaluated *in vivo*. The ileal relative abundance of *C. perfringens* in the BL-fed birds was significantly lower than that in the SNE-infected birds (*P <*0.05, **Figure 2**). To identify and validate the inflammation caused by SNE and ameliorated by probiotic feeding, we compared the pathological damage of the ileum and liver in the NC, PC, and BL groups. Histological examination revealed that the ileum tissue structure of the NC group was normal (**Figure 2**). In the PC group, the lamina propria of ileum showed hyperemia, a large number of lymphocytes were infiltrated, and intestinal epithelial cells were necrotic and exfoliated.

In the BL group, the damage of ileum was alleviated, and the lamina propria was slightly congested. No obvious pathological lesions were found in the liver tissue of the NC group. Severe pathological damage was detected in broilers infected with SNE, i.e., obvious hepatocyte swelling and granular degeneration. Probiotic *B. licheniformis* H2 administration markedly ameliorated the pathological damage; mild granular degeneration was found in the BL group.

### Probiotic H2 Enhances the Intestinal Barrier Function and Epithelial Renewal

**Figure 3:**
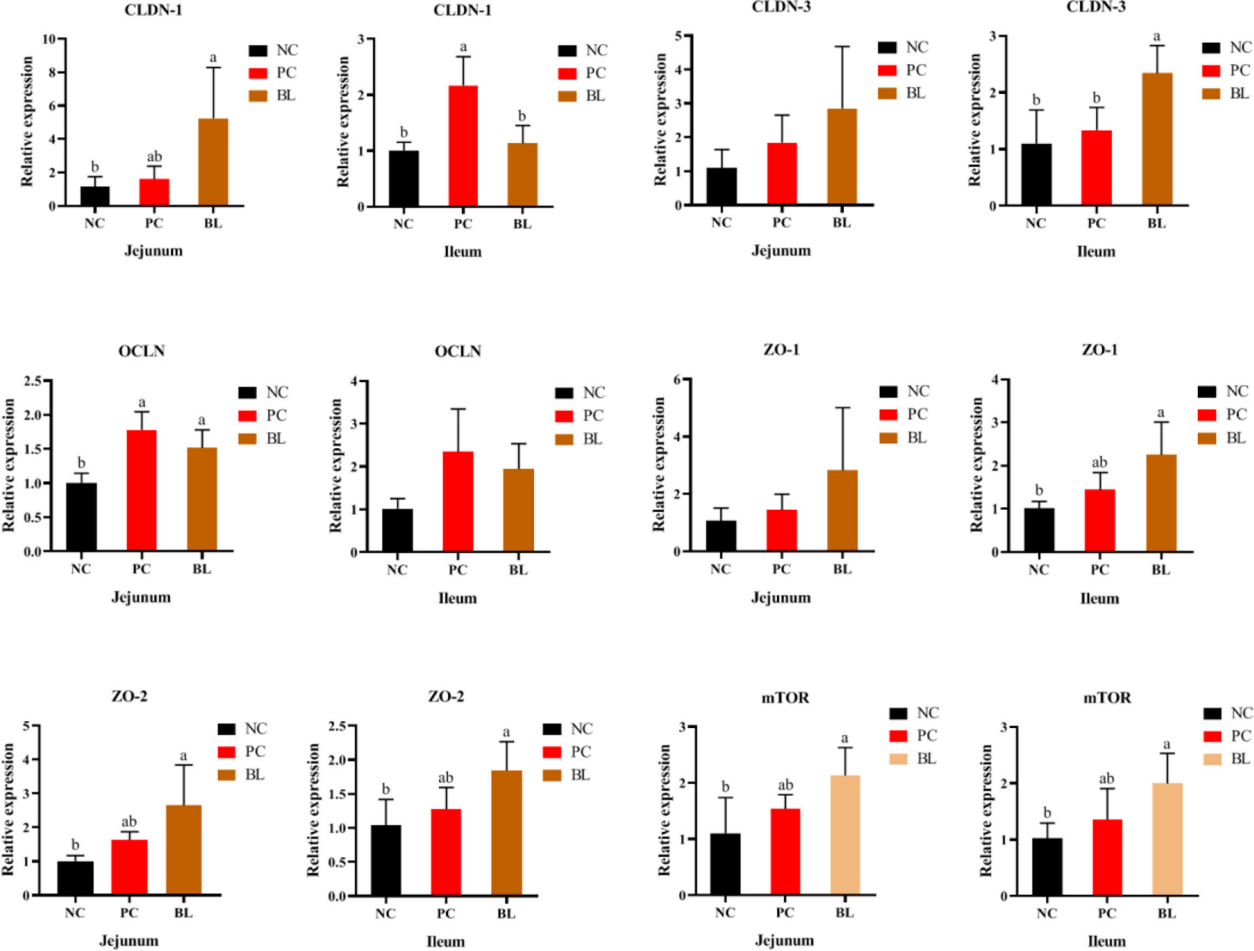
Relative mRNA expressions of mTOR, CLDN-1, CLDN-3, OCLN, ZO-1, and ZO-2 in the jejunum and ileum of broiler chickens (n = 6, mean with SD). a, b means with different letters are significantly different (*P <*0.05). One-way ANOVA and adjustment for multiple comparisons were conducted.

Liver inflammation indicated destruction of intestinal epithelial barrier. Therefore, we further analyzed the effect of H2 on intestinal barrier in broilers. The CLDN-1 gene expression was higher in the jejunum in the BL group compared with the NC and PC groups (**Figure 3**). The expression levels of CLDN-3, ZO-1, and ZO-2 in the ileum and jejunum of the BL group were higher than those of the NC and PC groups. Our data indicated that oral administration of H2 significantly upregulated the expression of tight junction proteins, suggesting a promotion of gut integrity. Furthermore, mTOR expression was significantly enhanced after probiotic feeding (*P <*0.05), indicating an accelerated intestinal epithelial cell renewal.

### Probiotic H2 Ameliorates the Inflammatory Response and Increases Immunity to SNE-Infected Broilers

**Figure 4:**
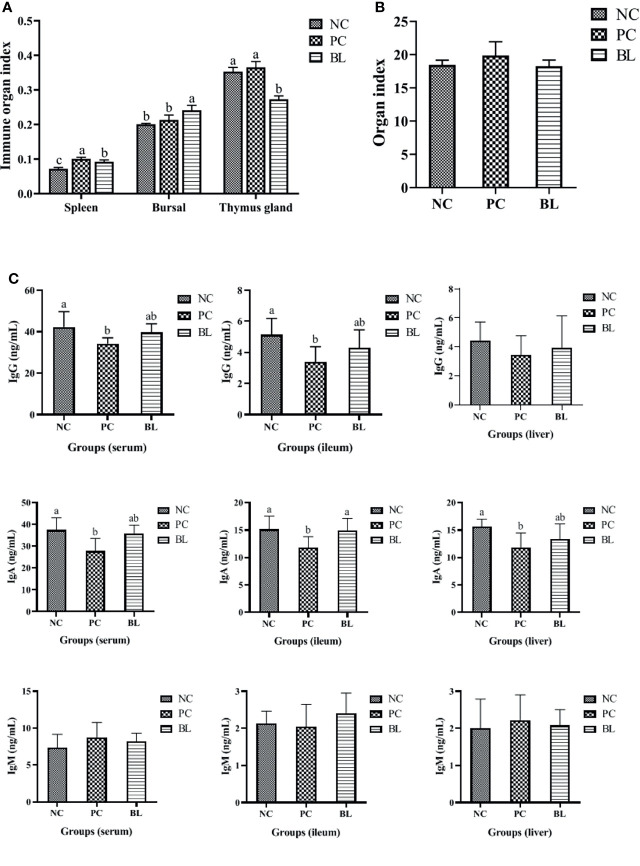
Probiotic H2 ameliorates the inflammatory response and increases the immunity of SNE-infected broilers. **(A, B)** Comparison of organ indexes from thymus gland, bursa, spleen, and liver (n = 4, mean with SD). **(C)** Protein contents of IgG, IgA, and IgM were determined in the serum, ileum, and liver of broiler chickens (n = 6, mean with SD). a, b means different letters are significantly different (*P <*0.05). One-way ANOVA and adjustment for multiple comparisons were conducted.

In general, destroyed intestinal barrier (“leaky gut”) and increased intestinal permeability could enable intestinal bacteria or bacterial products/toxins to harm the liver through the portal vein, thus promoting systemic inflammation (33). The H2-fed birds had a significant increased immune organ index of bursal (*P <*0.05, **Figure 4**). The liver organ index of broilers increased in the SNE-infected birds (*P >*0.05), whereas this was reduced in the H2-fed birds (*P >*0.05), indicating an occurrence of inflammation caused by SNE, which was alleviated by probiotics (**Figure 4**).

**Figure 5:**
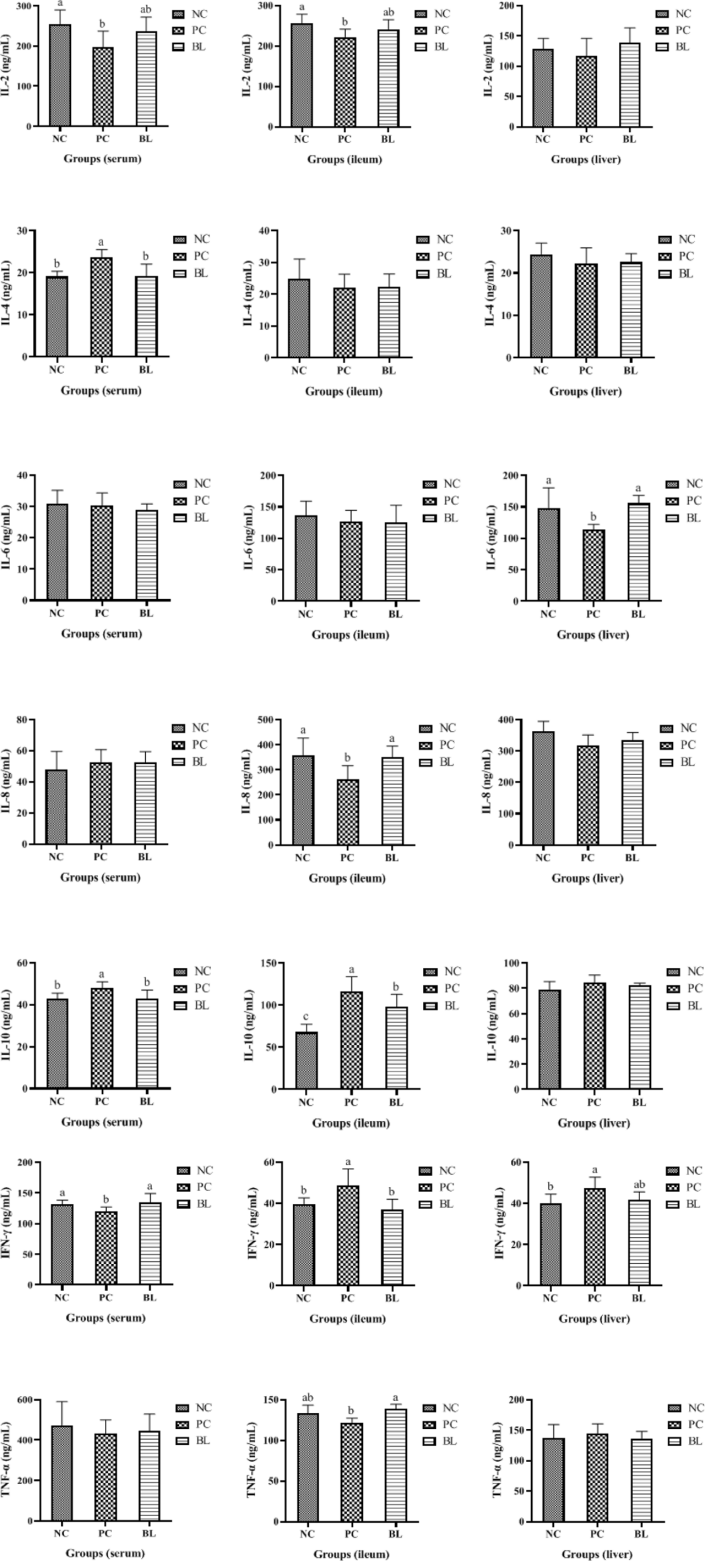
Protein contents of IL-2, IL-4, IL-6, IL-8, IL-10, IL-17, IFN-γ, and TNF-α were determined in the serum, ileum, and liver of broiler chickens (n = 6, mean with SD). a, b means those with different letters are significantly different (*P <*0.05). One-way ANOVA and adjustment for multiple comparisons were conducted.

To assess the immune response during SNE with or without probiotic treatment, the levels of cytokines and immunoglobulins in the serum, ileum, and liver were detected (**Figures 4**, **5**). The contents of IL-2 and IFN-γ in the serum, IL-2 and IL-8 in the ileum, and IL-6 in the liver were significantly decreased in the PC group compared with the NC group (*P <*0.05), whereas the contents of IL-4 and IL-10 in the serum, IL-10 and IFN-γ in the ileum, and IFN-γ in the liver were significantly increased (*P <*0.05). No significant difference was found between the NC and BL groups in terms of the levels of IL-2, IFN-γ, IL-4, and IL-10 in the serum, IL-2, IFN-γ, and IL-8 in the ileum, and IL-6 and IFN-γ in the liver (*P >*0.05). Moreover, the serum and ileum contents of IgG in the PC group were significantly decreased compared with the NC group (*P <*0.05), whereas the contents of IgG were enhanced by H2 (*P >*0.05). The serum, ileum, and liver contents of IgA in the PC group were significantly decreased compared with the NC group (*P <*0.05), whereas the contents of IgA were enhanced by H2. Improvement in the contents of IgG and IgA was detected in the BL group, suggesting a boosted immunity after H2 feeding.

### Probiotic H2 Promotes Growth Metabolism and Nutrient Transport

**Figure 6:**
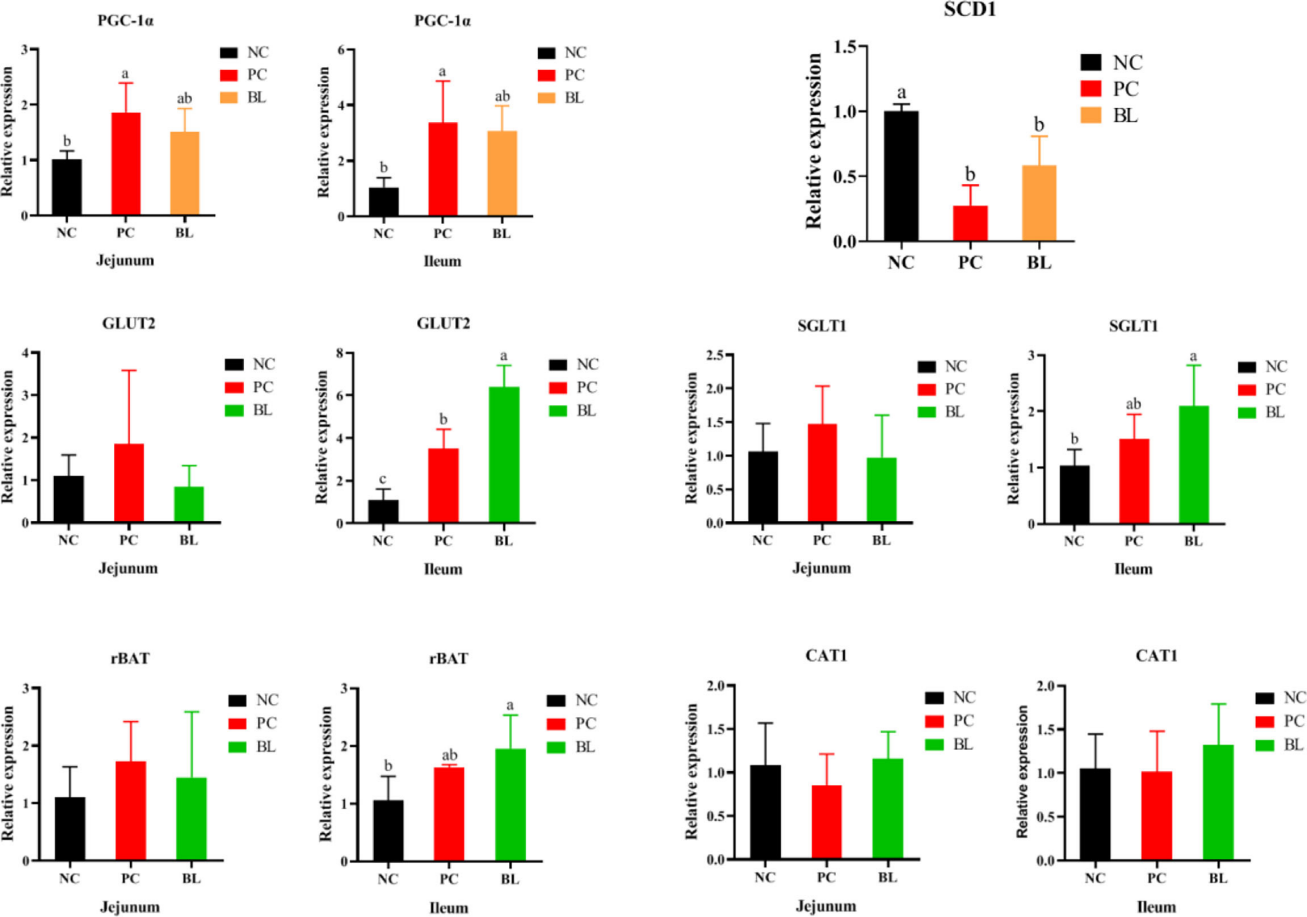
Relative mRNA expressions of PGC-1α, SCD1, GLUT2, SGLT1, rBAT, and CAT1 of broiler chickens (n = 6, mean with SD). a, b means those with different letters are significantly different (*P <*0.05). One-way ANOVA and adjustment for multiple comparisons were conducted.

Immune responses are a process of energy consumption that diverts nutrients from growth and subsequently reduces growth performance (34). Our data showed that the oral administration of H2 contributed to the reduction of PGC-1α expression in the jejunum and ileum, indicating a reduction in energy consumption (*P <*0.05, **Figure 6**). After H2 feeding, SCD1 gene expression markedly increased, indicating an improvement in metabolic dysfunction and hepatocyte apoptosis (*P <*0.05, **Figure 6**). Next, we investigated the effect of H2 on intestinal nutrient absorption. The oral administration of H2 significantly increased the expressions of glucose and amino acid transporter genes (GLUT2, SGLT1, and rBAT) in the ileum (*P <*0.05, **Figure 6**). Interestingly, the opposite result was detected in the jejunum. These findings revealed that H2 significantly increased the expression levels of glucose and amino acid transporter gene expressions, which were related to nutrient absorption.

### Liver Transcriptional Changes Induced by SNE and Probiotic H2 Treatment

**Figure 7:**
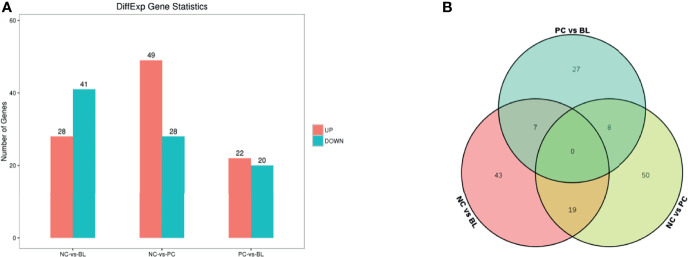
Identification of differentially expressed genes in the liver. **(A)** RNA-Seq analysis reveals differentially expressed genes between the NC and the BL groups, the NC and the PC groups, and the PC and the BL groups. Red and blue colors represent up and downregulated transcripts, respectively. **(B)** Venn diagram for the DEGs.

By using RNA-Seq, we investigated the liver transcriptional alterations associated with SNE-infected broilers with or without H2. As shown in **Figure 7A**, 77, 69, and 44 differentially expressed genes (DEGs; FDR <0.05 and |log_2_FC| >1) were discovered in three comparison levels, namely, PC group vs NC group, BL group vs NC group, and BL group vs PC group, respectively. The Gene Ontology (GO) enrichment analysis suggested that the DEGs might be involved in immune response, immunoglobulin binding, MHC protein binding, antigen processing and presentation, antigen binding, cell activation involved in immune response, glucose transport, lipid metabolic, ATP biosynthetic process, ATP metabolic process and cell cycle G1/S phase transition (**Appendix Tables S3–5**). The Kyoto Encyclopedia of Genes and Genomes (KEGG) pathway enrichment analysis suggested that the DEGs may be involved in the regulation of ErbB signaling pathway, metabolic pathways, fatty acid degradation, fatty acid metabolism, synthesis and degradation of ketone bodies, TGF-beta signaling pathway, PPAR signaling pathway, Jak-STAT signaling pathway, MAPK signaling pathway, and cell cycle (**Appendix Table S6** and **Figure S1**). Venn diagram analysis suggested that three genes (MHCIY, SCD, and ALPP) correlated strongly with immune response and metabolic process were significantly expressed in the NC group vs the PC group, and the PC group vs the BL group, but no significant expression was found in the NC group vs the BL group (**Figure 7B** and **Appendix Table S7**). Four genes (LOC771804, FCER1G, LOC107050390, and CHIR-B3) correlated strongly with immune response were significantly expressed in the NC group vs the PC group, but not in the NC group vs the BL group and the PC group vs the BL group (**Figure 7B** and **Appendix Table S7**).

### Probiotic H2 Normalizes the Intestinal Microbiota Composition

**Figure 8:**
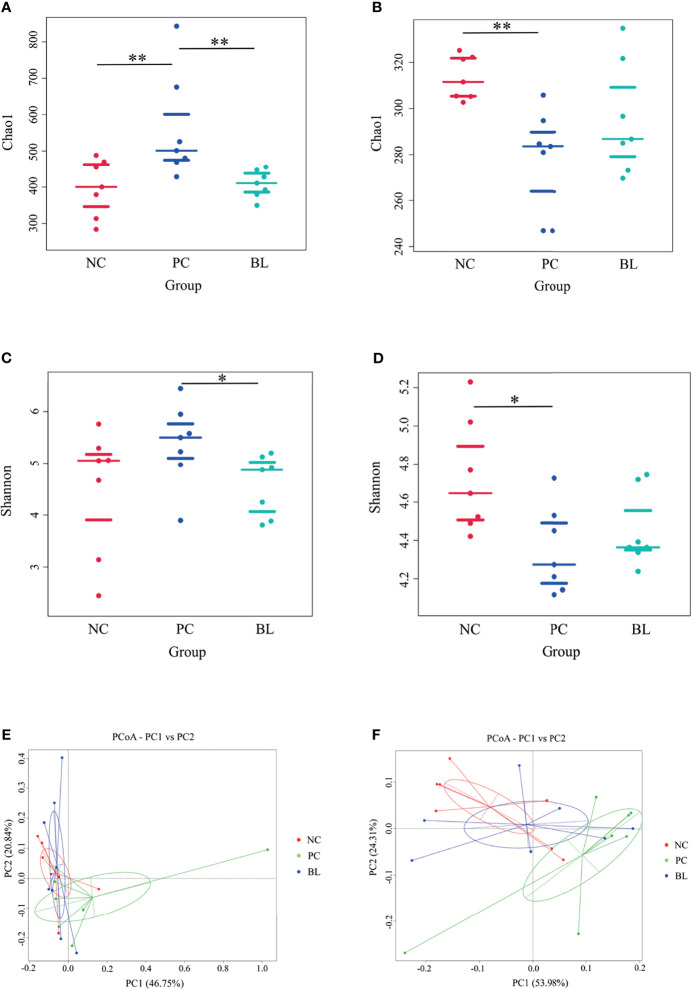
Bacterial alpha diversity and principal-coordinate analysis (PCoA) in the ileum **(A, C, E)** and cecum **(B, D, F)**. *P < 0.05, **P < 0.01.

**Figure 9:**
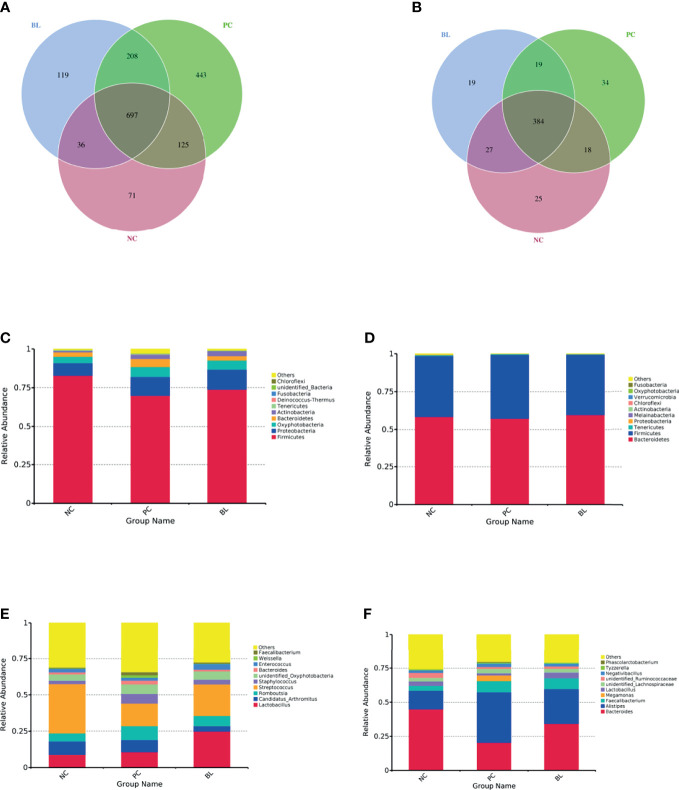
Venn diagrams for OTU and bacterial taxonomic compositions at the levels of phylum and genus in the ileum **(A, C, E)** and the cecum **(B, D, F)**.

The structure and functionality of the gut microbiota, namely, intestinal barrier integrity, nutrient absorption, metabolism, and immunity, play a crucial role in poultry health (25, 26). We then investigated which components of the gut microbiota of broiler chickens were involved in the amelioration of SNE by orally administered H2 using 16S rDNA amplicon sequencing analysis. The rarefaction curves suggested that almost all bacterial species were identified in ileal and cecal samples of birds (**Appendix Figure S2**). Chao1 and Shannon indices showed that bacterial species composition and diversity of ileum and cecum in BL and NC groups were more similar (**Figure 8**). Other indices of alpha diversity, namely, ACE, Observed-species, Simpson, and Good-coverage also showed similar changes (**Appendix Figure S3**). The beta diversity analysis based on weighted unifrac distances further showed that gut microbiota of the NC group had a higher similarity with that of the BL group, but had a lower similarity with that of the SNE group. Venn diagrams showed the unique and shared intestinal OTUs of the different groups in the ileum and cecum. The number of SNE-induced unique OTUs in both ileum and cecum decreased after *B. licheniformis* treatment (**Figure 9**). Intestinal microbial taxonomic analysis indicated that SNE infection resulted in an alteration in the microbiota composition of broiler chickens, and *B. licheniformis* treatment rescued the SNE-induced trend in microbiota composition (**Figure 9**). At the phylum level of microbiota in the ileum, the relative abundance levels of *Firmicutes*, *Proteobacteria*, and *Chloroflexi* decreased in the PC group compared with the NC group, whereas they increased in the BL group compared with the PC group. Moreover, the relative abundance levels of *Oxyphotobacteria*, *Bacteroidetes*, *Deinococcus-Thermus*, and *Fusobacteria* increased in the PC group compared with the NC group, whereas they decreased in the BL group compared with the PC group. *Firmicutes* and *Bacteroidetes* were the most predominant phyla in microbial communities across cecum. At the phylum level of microbiota in cecum, the relative abundance levels of *Bacteroidetes*, *Tenericutes*, *Proteobacteria*, and *Melainabacteria* decreased in the PC group compared with the NC group, whereas they increased in the BL group compared with the PC group. Moreover, the relative abundance of *Firmicutes* increased in the PC group compared with the NC group, whereas they decreased in the BL group compared with the PC group. At the genus level of microbiota in the ileum, the relative abundance levels of *Streptococcus* and *Enterococcus* decreased in the PC group compared with the NC group, whereas they increased in BL group compared with the PC group. The relative abundance levels of *Romboutsia*, *Staphylococcus*, *unidentified_Oxyphotobacteria*, *Bacteroides*, *Weissella*, and *Faecalibacterium* increased in the PC group in comparison with the NC group, whereas they decreased in the BL group in comparison with the PC group. Moreover, *Lactobacillus* increased and *Candidatus_Arthromitus* decreased with the probiotic diet in comparison with other groups. At the genus level of microbiota in cecum, the relative abundance levels of *Bacteroides*, *Lactobacillus*, *unidentified_Ruminococcaceae*, and *Tyzzerella* decreased in the PC group in comparison with the NC group, whereas they increased in the BL group in comparison with the PC group. The relative abundance levels of *Alistipes*, *Megamonas*, *unidentified_Lachnospiraceae*, *Negativibacillus*, and *Phascolarctobacterium* increased in the PC group in comparison with the NC group, whereas they decreased in the BL group in comparison with the PC group. Metastats analysis exhibited significant variability in ileal and cecal bacterial composition at the phylum and genus levels (**Appendix Figure S4**). SNE infection resulted in the remarkable reduction of *unidentified_Ruminococcaceae* and *Hydrogenoanaerobacterium* and the remarkable increase of *Candidatus soleaferrea* in intestinal microbiota. The abundance levels of *unidentified_Ruminococcaceae* and *Hydrogenoanaerobacterium* increased after H2 feeding. LEfSe analysis revealed that the PC group in the ileum had more differential biomarkers, followed by the BL and NC groups (**Appendix Figure S5**). Bacterial co-occurrence network also revealed that the number of ileal genera that was directly correlated with *Bacillus* in the PC group was more than those in the NC group and BL groups (**Appendix Figure S6**). These results suggested the higher similarity in bacterial community between the NC and BL groups.

### Probiotic H2 Normalizes the Function of Intestinal Bacteria

**Figure 10:**
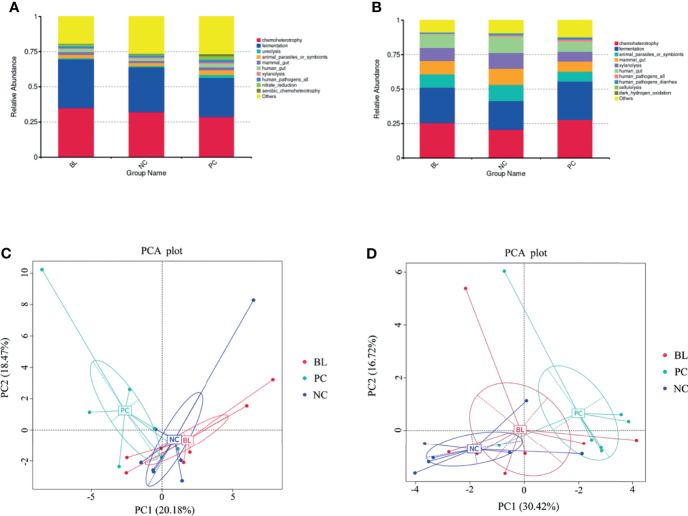
Probiotic H2 normalizes the function of the ileum **(A, C)** and the cecum bacteria **(B, D)**.

Next, we analyzed the function of gut microbiota in the broiler chickens. The prediction of the function of gut microbiota showed the high similarity between the NC and BL groups, whereas both the NC and the BL groups were distinguishable from the PC group (**Figure 10**). This trend was reflected in chemoheterotrophy, fermentation, ureolysis, mammal gut, xylanolysis, animal parasites or symbionts, and aerobic chemoheterotrophy functions. The results of principal component analysis also confirmed this trend.

### Changes in Gene Expression Profiles are Correlated With Microbiota Alterations

**Figure 11:**
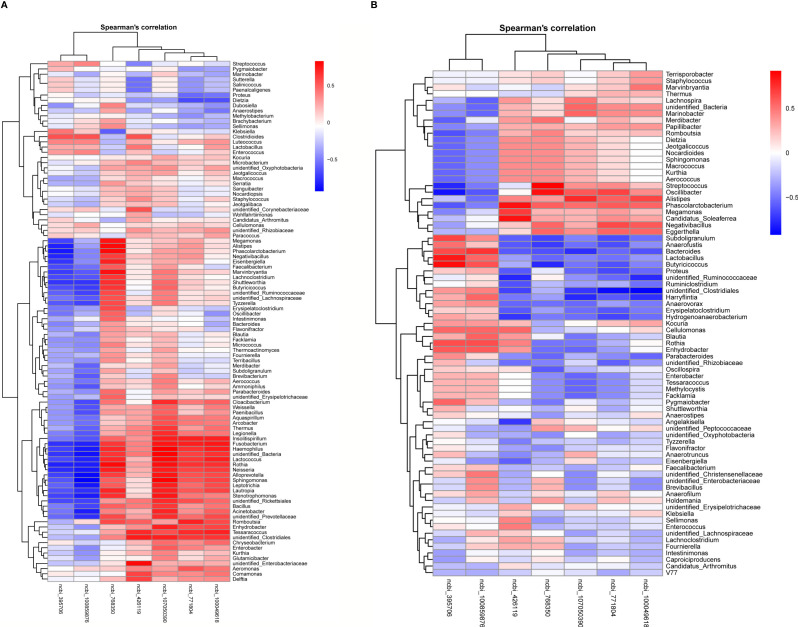
Heatmaps of Spearman correlation analyses between the abundance of top 100 bacterial genera and two metabolic and three immune genes in the ileum **(A)** and the cecum **(B)**.

Next, we investigated the potential association between gut microbiota and hepatic gene expression by the Spearman correlation coefficients. Seven differentially expressed genes were involved in metabolic and immune responses, as concluded from results of the liver transcriptome. According to the results of 16S rDNA amplicon sequencing, the top 100 abundant bacterial genera were screened out. The correlations between bacterial genera and hepatic genes with altered expression levels are represented in heatmaps (**Figure 11**). Interestingly, high positive correlations were found between bacterial genera and immune genes, high negative correlations between bacterial genera and metabolic genes simultaneously. Our results demonstrated that the numbers of *Bacteroides*, *Lactobacillus*, *unidentified_Ruminococcaceae*, *Erysipelatoclostridium*, *Hydrogenoanaerobacterium*, *Unidentified clostridiales*, and *Subdoligranulum* in the cecum decreased in the SNE group compared with the NC group, whereas they increased in the BL group compared with the SNE group. These numbers were positively correlated with metabolic gene expression and negatively correlated with immune gene expression. In addition, *Alistipes*, *Megamonas*, *Negativibacillus*, *Candidatus soleaferrea*, *Romboutsia*, and *Phascolarctobacterium* increased in the SNE group compared with the NC group, whereas they decreased in the BL group compared with the SNE group. They were positively correlated with immune gene expression, and negatively correlated with metabolic gene expression. In the ileum, *Romboutsia*, *Weissella*, *Faecalibacterium*, *Rothia*, *Insolitispirillum*, *Megamonas*, *Eisenbergiella*, *Unidentified bacteria*, *Fusobacterium*, and *Stenotrophomonas* increased in the SNE group compared with the NC group, whereas they decreased in the BL group compared with the SNE group. They were positively correlated with immune gene expression, and negatively correlated with metabolic gene expression. The correlation analyses between these genera in the ileum and cecum and other indicators unveiled they also altered gut immunity and metabolism (**Appendix Figure S7**).

## Discussion

A steady increase in the population of the world has led to the growth in demand for poultry products (35, 36). SNE significantly impacts the attempts to increase global poultry production, leading to the great difficulty in meeting the global demand for animal protein (35, 36). In the past, using antibiotics to manage gut microbiota and control diseases was the norm. Given that antibiotics have detrimental impacts on the environment and food chain worldwide, alternatives to antibiotics are urgently needed to prevent SNE in poultry farming (37). Probiotics are known to improve the gut health and growth performance of the host and have received considerable attention over the last decade (38). Gut health broadly includes normal intestinal physiology, complete intestinal epithelial barrier, efficient immune responses, metabolism and energy balance, sustained inflammatory balance, high nutrient absorption efficiency, and especially, healthy microbiota (35). However, the precise mechanism of probiotic action and which gut microbes confer this efficacy remain largely unknown.

Strong genetic selection for productive performance of animals adversely affects animal welfare and natural immunity, thereby also decreasing disease tolerance (39). Currently, disease control and reasonable production costs have become issues that needed to be addressed in the poultry industry (39). The support of gut health by probiotics is crucial for maintaining optimum performance and controlling diseases of broiler chickens (35). H2 was identified as a promising antibiotic alternative for preventing SNE-induced inhibition of broiler performance (29). By assessing the abundance of *C. perfringens in vivo*, we found that the relative abundance of *C. perfringens* was significantly decreased after H2 feeding. We also observed that probiotic H2 administration markedly ameliorated the pathological damage to the ileum and liver. These results indicated that probiotic H2 could suppress SNE-induced inflammation in broiler chickens.

Intestinal barrier plays an important role in preventing liver exposure to intestinal bacteria or bacterial products/toxins (15). Tight junctions (TJ) are a multi-protein complex that tightly controls paracellular permeability across the intestinal epithelium, which can be used as a marker for gut health and epithelial barrier integrity (40, 41). *In vivo* and *in vitro* studies have shown that the loss of the tight junction integrity greatly leads to a phenomenon commonly known as “leaky gut” (40, 41). This intestinal barrier dysfunction exposes the liver to bacterial components and metabolites and intestinal bacteria, leading to liver inflammation (42, 43). Enteric pathogens can reportedly destroy tight junctions and increase paracellular permeability. For example, *C. perfringens* enterotoxin (CPE) produced by CP uses TJ proteins (certain members of the claudin family) as cellular receptors; these are responsible for increased paracellular permeability (42, 43). SNE drove intestinal barrier dysfunction and induced liver inflammation (32, 44, 45). These negative influences were effectively inhibited by probiotics (i.e., *Lactobacillus*, *Bacillus*, and *C. butyricum*) (32, 44, 45). Consistent with these studies, our findings indicated that H2 was involved in the enhancement of tight junction protein expression. Intestinal epithelium has rapid self-renewing and powerful regenerative ability after infection and inflammation injury (46, 47). The renewal of intestinal epithelial cells along the crypt-villus axis is regulated by the mechanistic target of rapamycin (mTOR) signaling pathway (46, 47). Our study demonstrated that the expression level of mTOR was significantly increased by H2, suggesting an accelerated intestinal epithelial cell renewal. The capacity of mTOR signaling pathway to regulate intestinal epithelial cell renewal may be carried out partly through its effect on the antioxidant capacity and protein synthesis of intestinal epithelial cells (46). Our previous studies have shown that H2 exerted a positive effect on the antioxidant capacity of broiler chickens, which ensured that the meat quality of broilers was not damaged by oxidative stress (29).

Intestinal barrier dysfunction contributes to disease development, thereby increasing the susceptibility to chronic inflammation, malabsorption, and systemic infection (15). The modification of the organ index demonstrated that birds might be suffering from SNE infection. H2 could change the levels of cytokines and immunoglobulins in serum, ileum, and liver, demonstrating alleviated inflammation and boosted immunity in birds. Immune response is an energy-consuming process (48). The activation of the immune function requires the synthesis of many new molecules and undertakes numerous cellular tasks, and it must occur rapidly (53]. Therefore, the immune system diverts nutrients from growth to ensure sufficient energy for an effective response (resistance), subsequently reducing growth performance (32, 48). In this study, we demonstrated that H2 significantly decreased the expression of PGC-1α associated with cell energy metabolism. SCD1 plays a key role in general metabolism and nutrition (49). We observed that SCD1 gene expression was markedly increased after H2 feeding, indicating a positive effect on metabolic and nutritional regulation. Moreover, broilers are fast growing due to the great potential of intestinal epithelium for nutrient absorption and efficient conversion of nutrient to muscle (50). In the present study, we observed that H2 significantly increased the expression levels of glucose and amino acid transporters associated with nutrient absorption in the ileum. These results suggested that H2 significantly reduced the energy consumption of the immune response and optimized the nutrient absorption efficiency of the intestinal epithelium. The gene expression levels of glucose and amino acid transporters in the jejunum were contradictory. A possible explanation is that jejunum and ileum differed in terms of physiological structure and disease severity.

Gut–liver axis represents a close functional and physiological connection between the gut and the liver (51, 52). The liver receives about 70% of its blood supply from the portal vein outflowing from the intestine (51, 52). Therefore, the disruption of the intestinal barrier increases the risk of liver exposure to gut-derived products. Liver injury can lead to the impairment of the bile acid metabolism and the promotion of intestinal dysmotility and systemic inflammation, thereby resulting in gut dysbiosis, which in turn further exacerbates liver damage (51–53). Given that SNE induces injuries in the liver through the gut microbiota, we used RNA-Seq to compare gene expression in the liver of normal, SNE-infected, and H2-fed birds. Differentially expressed genes may be involved in immune response and metabolism-related pathways. Of note, we identified that the two genes (SCD and ALPP) correlated strongly with metabolic process, and one gene (MHCIY) correlated strongly with immune response; these were significantly expressed in the NC group vs the PC group and the PC group vs the BL group but not in the NC group vs the BL group. Four genes (LOC771804, FCER1G, LOC107050390, and CHIR-B3) correlated strongly with immune response were significantly expressed in the NC group vs the PC group, but not in the NC group vs the BL group, and the PC group vs the BL group. Our findings suggested that these seven genes may be regulated by probiotic H2 through unrecognized gut microbes.

Gut microbiota is the crucial organ that influences nutrient absorption, feed digestibility, energy utilization, and productivity in poultry (35). The early colonization and establishment of gut microbiota in chicks can vary the morphology and physiology of the gut and the susceptibility to infectious diseases (54). Therefore, one of the advantages of probiotics is that early probiotic supplementation is conducive to shaping healthy intestinal microbiota and enhancing disease resistance of broiler chickens (55, 56). In this study, we found that the composition and diversity of gut microbiota in the BL group had a high similarity with those in the NC group but a low similarity with the SNE group. Also, the prediction results of gut microbiota function showed the high similarity between the NC and the BL groups, whereas both the NC and the BL groups were distinguishable from the PC group. Additionally, the stage of liver injury reportedly correlates closely with the severity of gut dysbiosis (57). To investigate the relationship between altered microbiota and liver gene expression, we compared normal broilers and SNE-infected broilers with or without H2 using the Spearman correlation coefficients. Interestingly, we found a high positive correlation between a few bacterial genera and immune genes/metabolic genes, whereas a high negative correlation was found between a few bacterial genera and immune genes/metabolic genes simultaneously. For example, the relative abundance of butyrate-producing bacteria *Erysipelatoclostridium*, *Subdoligranulum*, and *unidentified_Ruminococcaceae* in the cecum of BL-fed birds were higher than that of SNE-infected birds, suggesting that H2 inhibited the negative effects caused by SNE. They showed high and positive correlation with liver metabolic genes and high and negative correlation with liver immune genes, indicating that they are beneficial to the host by promoting growth and metabolism and by reducing immune responses. Similarly, *Lactobacillus* has probiotic activities, namely, immunomodulation and pathogen inhibition, and such activities increased after H2 addition (58). *Alistipes* is highly associated with dysbiosis and disease, such as non-alcoholic steatohepatitis and liver fibrosis, which are reduced after H2 addition (59). These results suggested that H2 suppresses immune response and promotes growth and metabolism by regulating liver gene expression, which relies on gut microbiota.

## Conclusions

SNE can cause inflammation in the gut and liver of broiler chickens, destroy the intestinal barrier, and result in gut dysbiosis, thereby reducing production efficiency. These negative effects were effectively suppressed by probiotic H2. Probiotic feeding improved intestinal barrier, and enhanced nutrient absorption efficiency. Additionally, probiotic feeding regulated liver gene expression to suppress immune response and promotes growth and metabolism relies on the gut microbiota. These results suggest that H2 may serve as an antibiotic alternative for preventing SNE in poultry farming.

## Data Availability Statement

The datasets presented in this study can be found in online repositories. The names of the repository/repositories and accession number(s) can be found in the article/**Supplementary Material**. All raw data of liver transcriptome RNA sequencing (Accession number: PRJNA719705) and 16S rRNA amplicon sequencing (Accession number: PRJNA721597) are available at NCBI SRA database.

## Ethics Statement

All animal experiment procedures in this study were conducted in accordance with the guidelines of the Animal Welfare Act and all procedures and protocols were approved by the Institutional Animal Care and Use Committee of the Sichuan Agricultural University (approval number: SYXKchuan2019-187). All the efforts were made to minimize the suffering of the animals.

## Author Contributions

YZhao, YZeng, and XN conceived the study and designed the project. YZhao, DZ, WH, NS, and XJ carried out the experiments. YZeng wrote the main manuscript text. YZhao and NS analyzed the data. All authors listed have made a substantial, direct, and intellectual contribution to the work and approved it for publication.

## Funding

This work was supported by the National Natural Science Foundation of China (31970503) and the Sichuan Science and Technology Program (2021YFH0097, 2018HH0103).

## Conflict of Interest

Author HY and LL were employed by the Chengdu Slan Biotechnology Co., Ltd. Author HL was employed by the Qingdao Vland Biotech Inc. The remaining authors declare that the research was conducted in the absence of any commercial or financial relationships that could be construed as a potential conflict of interest.

## Publisher’s Note

All claims expressed in this article are solely those of the authors and do not necessarily represent those of their affiliated organizations, or those of the publisher, the editors and the reviewers. Any product that may be evaluated in this article, or claim that may be made by its manufacturer, is not guaranteed or endorsed by the publisher.

## Abbreviations

SNE, Subclinical necrotic enteritis; NE, Necrotic enteritis; H2, *Bacillus licheniformis* H2; CP, *Clostridium perfringens*; OTU, Operational taxonomic units; PBS, Phosphate buffered saline; Claudin-1, CLDN-1; Claudin-3, CLDN-3; ZO-1, Zonula occluden-1; ZO-2, Zonula occluden-2; IL, interleukin; OCLN, Occludin; IgA, Immune globulin A; IgG, Immune globulin G; IgM, Immune globulin M; SCD1, Stearoyl-CoA desaturase 1, mTOR, mammalian target of Rapamycin; IFN-γ, Interferon-γ; TNF-α, Tumor necrosis factor-α; PGC-1α, peroxisome proliferator-activated receptor-gamma coactivator-1α; SGLT1, sodium-dependent glucose transporters-2; GLUT2, Glucose transporter type-2; rBAT, protein related to b^0,+^AT; CAT1, Cationic amino acid transporter-1.
