# Supplementary material for: Dietary Probiotic Supplementation Suppresses Subclinical Necrotic Enteritis in Broiler Chickens in a Microbiota-Dependent Manner
Source: Front Immunol. 2022 Mar 18;13:855426. doi: 10.3389/fimmu.2022.855426 (PMC8972058; doi:10.3389/fimmu.2022.855426)
Supplement: Supplementary file 1 [file DataSheet_1.pdf]

Supplemental Information

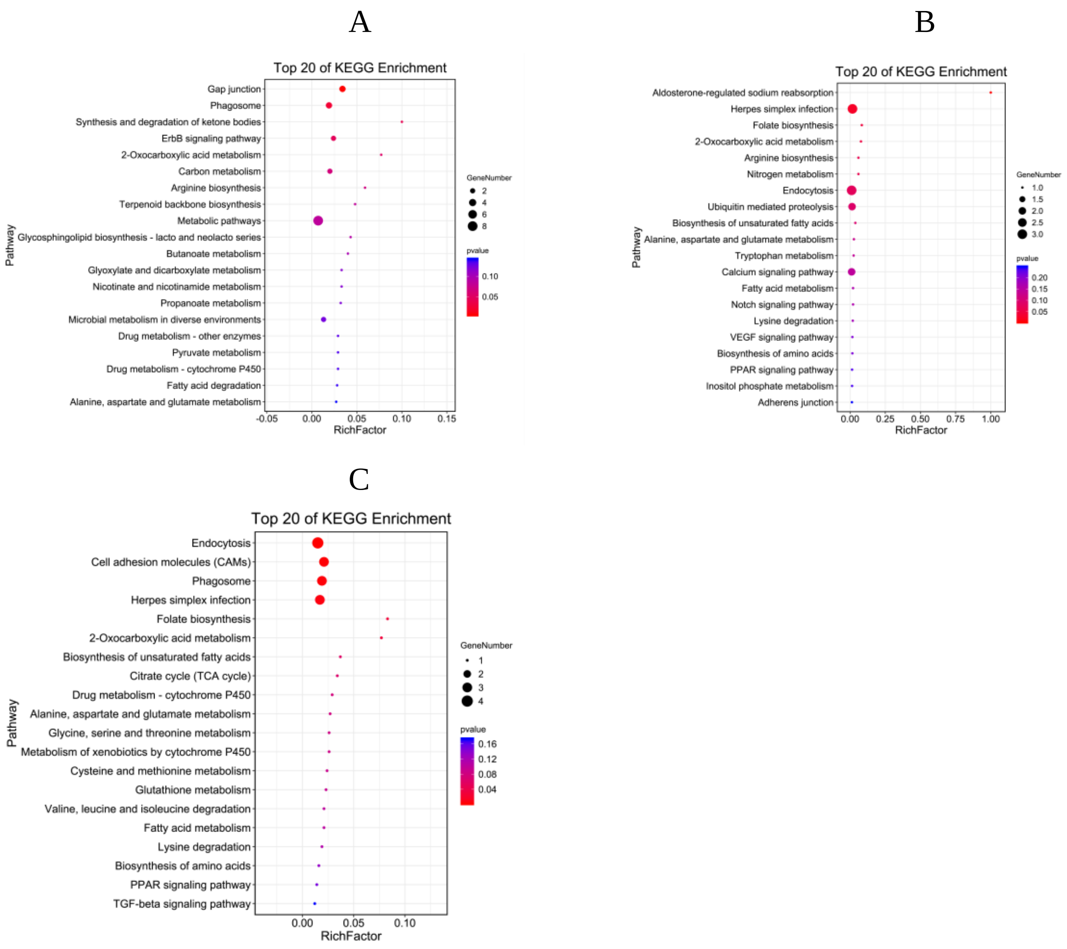

Figure 1 KEGG enrichment scatter plot of the DEGs. The horizontal axis represents the gene ratio, and the vertical axis represents the description of the KEGG pathway. The size of the dots indicates the number of enriched pathways, and the color of the dots corresponds to different *P* value ranges. (**A**)**:** NC vs BL, (**B**)**:** NC vs PC, (**C**)**:** PC vs BL.

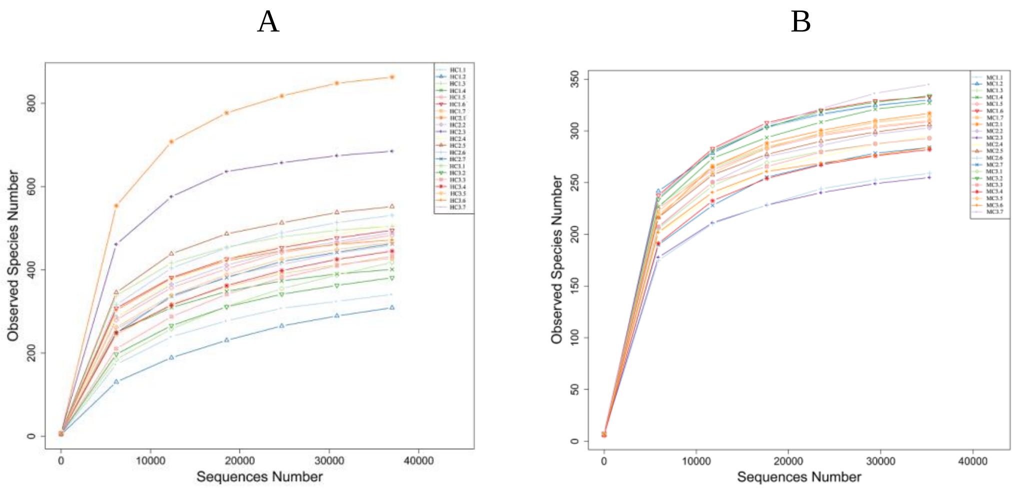

Figure 2 Rarefaction curves of bacterial sequencing in ileum **(A)** and cecum **(B)**.

A

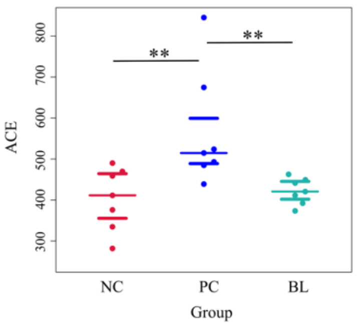
ACE
300
400
500
600
700
800
**
**
NC
PC
BL
Group

B

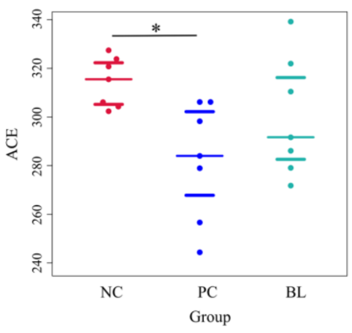
ACE
240
260
280
300
320
340
*
NC
PC
BL
Group

C

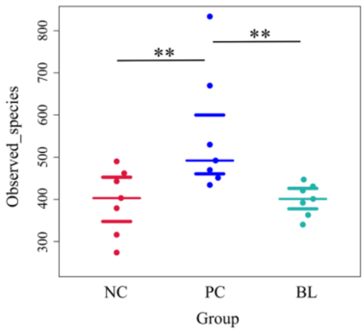
Observed_species
300
400
500
600
700
800
**
**
NC
PC
BL
Group

D

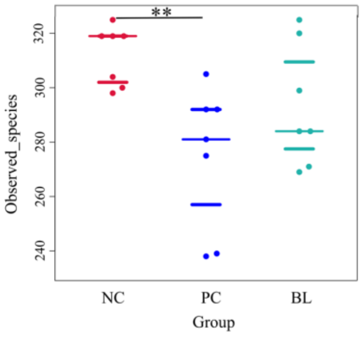
Observed_species
240
260
280
300
320
**
NC
PC
BL
Group

E

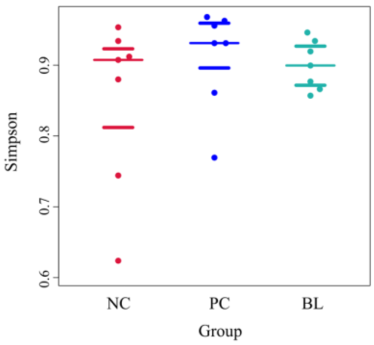
Simpson
0.6
0.7
0.8
0.9
NC
PC
BL
Group

F

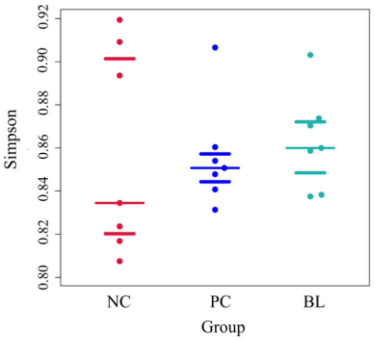
Simpson
0.80
0.82
0.84
0.86
0.88
0.90
0.92
NC
PC
BL
Group

G

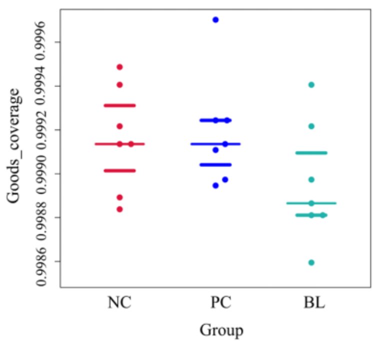
Goods_coverage
0.9986 0.9988 0.9990 0.9992 0.9994 0.9996
NC
PC
BL
Group

H

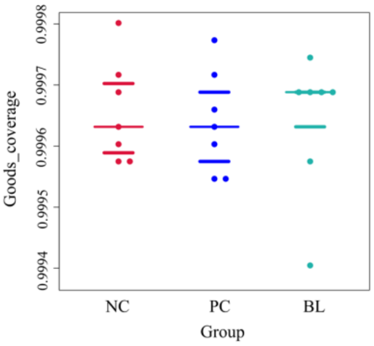
Goods_coverage
0.9994
0.9995
0.9996
0.9997
0.9998
NC
PC
BL
Group

Figure 3 The alpha diversity including ACE, Observed-species, Simpson, and Good-coverage in the ileal **(A, C, E, and G)** and cecal **(B, D, F, and H)** bacterial communities.

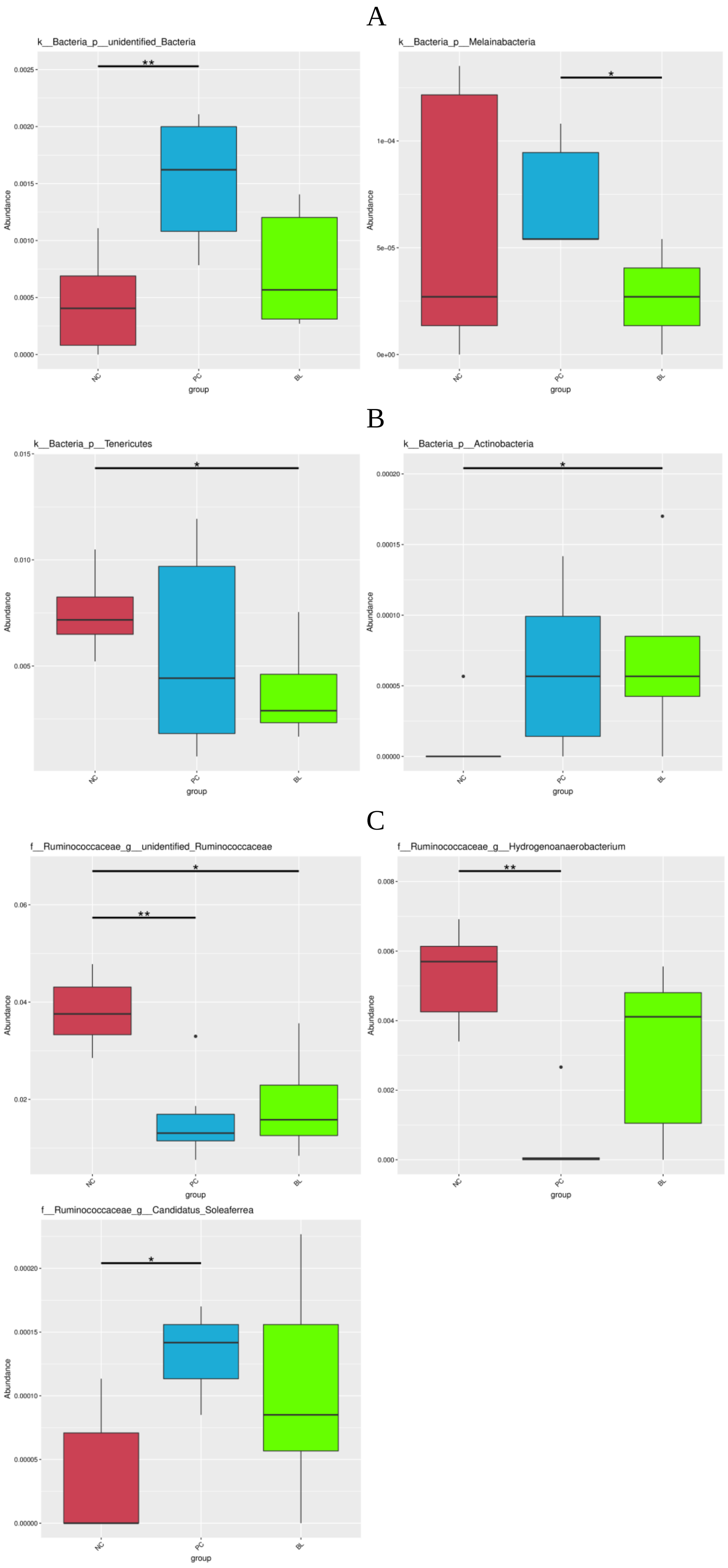

Figure 4 Metastats analysis was used to identify the significantly differential bacterial

phylum and genus. **(A):** The level of ileal bacterial phylum, **(B-C):** The level of cecal bacterial phylum and genus.

A

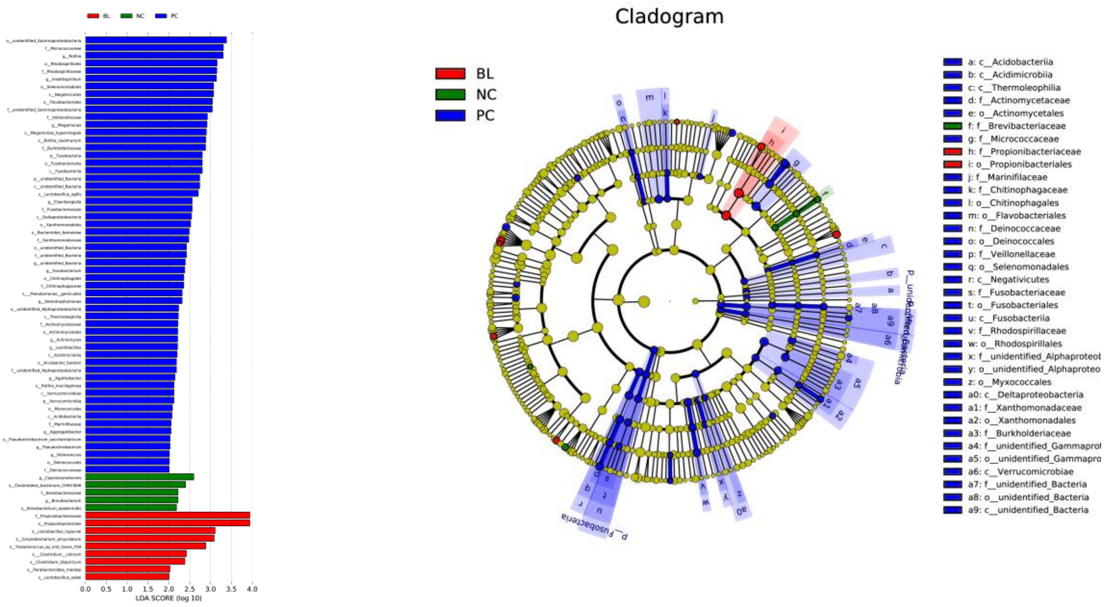

B

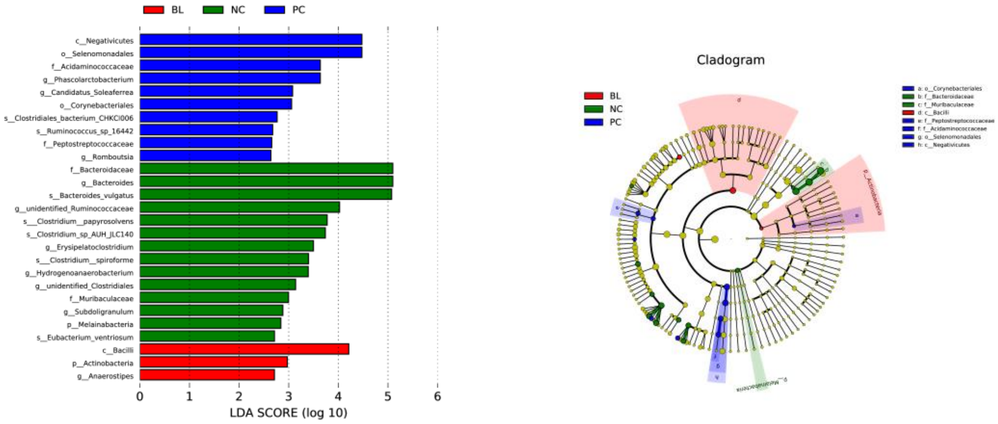

Figure 5 The LEfSe analysis of ileal **(A)** and cecal **(B)** bacterial communities.

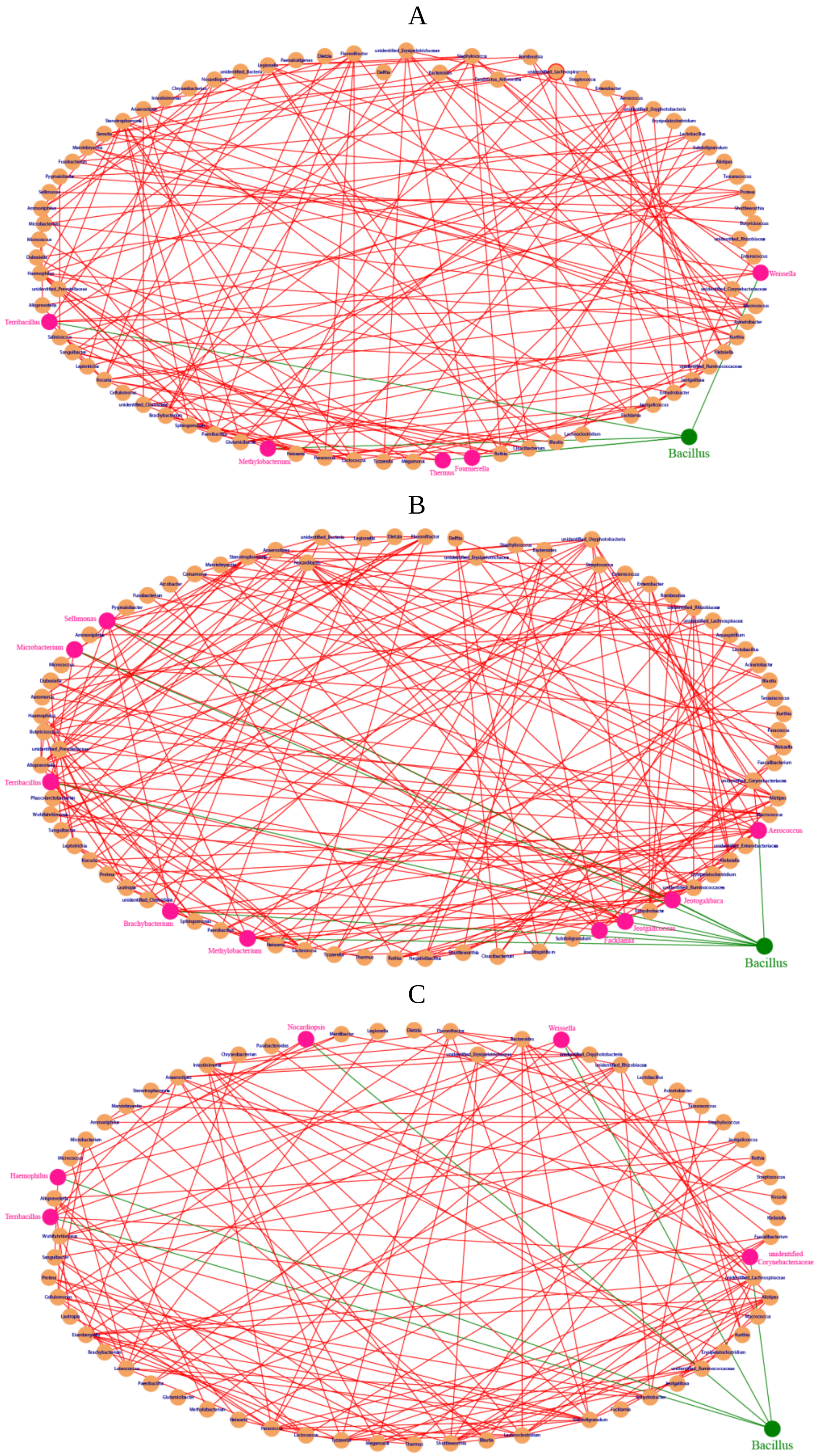

Figure 6 Bacterial co-occurrence network revealed the key role of *Bacillus*. (Correlation coefficient = 0.8). *Bacillus* are highlighted as green circle, while those

genera directly correlated with *Bacillus* are colored as purple. **(A):** NC group, (**B):** PC group, (**C):** BL group.

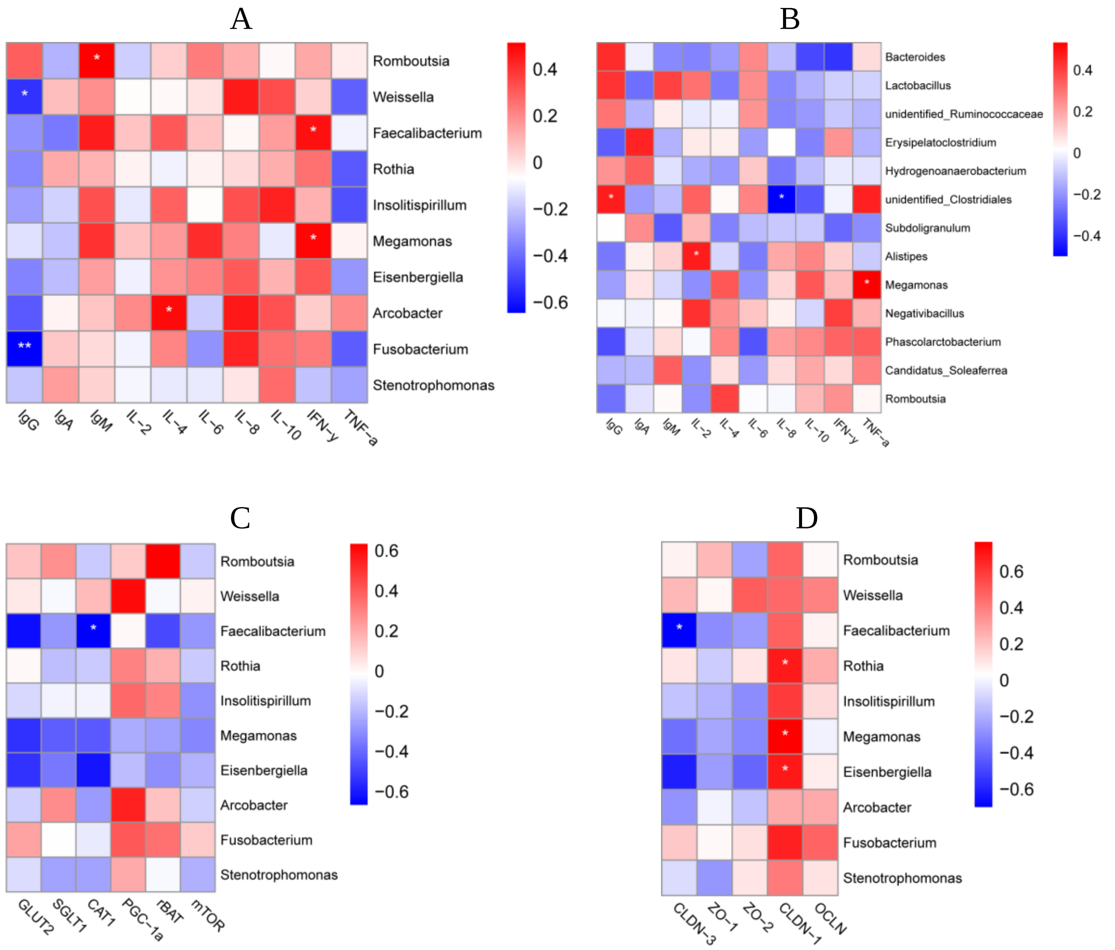

Figure 7 Heatmaps of Spearman correlation analyses between the abundance of ileum and cecum bacterial genera and indicators related to serum inflammation **(A and B)**, nutrient absorption **(C)**, and intestinal barrier function **(D)**. *: $P < 0.05$, **: $P < 0.01$.
